# Knockdown of LXRα Inhibits Goat Intramuscular Preadipocyte Differentiation

**DOI:** 10.3390/ijms19103037

**Published:** 2018-10-05

**Authors:** Yan Xiong, Qing Xu, Sen Lin, Yong Wang, Yaqiu Lin, Jiangjiang Zhu

**Affiliations:** 1 College of Life Science and Technology, Southwest Minzu University, Chengdu 610041, China; xiongyan0910@126.com (Y.X.); xq20170913@163.com (Q.X.); ls2349@163.com (S.L.); zhujiang4656@hotmail.com (J.Z.); 2 Key Laboratory of Qinghai-Tibetan Plateau Animal Genetic Resource Reservation and Utilization, Ministry of Education, Southwest Minzu University, Chengdu 610041, China; 3 Key Laboratory of Sichuan Province for Qinghai-Tibetan Plateau Animal Genetic Resource Reservation and Exploitation, Chengdu 610041, China

**Keywords:** goat, *Capra hircus*, LXRα, intramuscular adipocyte, intramuscular fat

## Abstract

Goat intramuscular fat (IMF) content is mainly determined by the processes of intramuscular preadipocytes adipogenic differentiation and mature adipocyte lipid accumulation. However, the underlying regulators of these biological processes remain largely unknown. Here, we report that the expression of Liver X receptor alpha (*LXRα*) reaches a peak at early stage and then gradually decreases during goat intramuscular adipogenesis. Knockdown of LXRα mediated by two independent siRNAs significantly inhibits intramuscular adipocytes lipid accumulation and upregulates preadipocytes marker- preadipocyte factor 1 (*pref1*) expression. Consistently, siRNA treatments robustly decrease mRNA level of adipogenic related genes, including CCAAT enhancer binding protein alpha (*Cebpα*), Peroxisome proliferator activated receptor gamma (*Pparg*), Sterol regulatory element binding protein isoform 1c (*Srebp1c*), Fatty acids binding protein (*aP2*) and Lipoprotein lipase (*Lpl*). Next, adenovirus overexpression of LXRα does not affect intramuscular adipocytes adipogenesis manifested by Oil Red O signal measurement and adipogenic specific genes detection. Mechanically, we found that both CCAAT enhancer binding protein beta (*Cebpβ*) and Kruppel like factor 8 (*Klf8*) are potential targets of LXRα, indicated by having putative binding sites of LXRα at the promoter of these genes and similar expression pattern during adipogenesis comparing to LXRα. Importantly, mRNA levels of *Cebpβ* and *Klf8* are downregulated significantly in goat LXRα knockdown intramuscular adipocyte. These results demonstrate that loss function of LXRα inhibits intramuscular adipogenesis possibly through down-regulation of *Cebpβ* and *Klf8*. Our research will provide new insights into mechanical regulation of goat IMF deposition.

## 1. Introduction

Intramuscular fat (IMF) content provides an indicator for marble score grading system of goat meat and high marbling cuts are consumption pursuits in many countries [1]. Thus, IMF is one of the most important traits for meat quality, appropriately elevated content of which improves meat color, water holding capacity, tenderness and flavor [2]. At the molecular level, IMF is mainly determined by the process of intramuscular preadipocytes differentiation into mature adipocytes [3]. Recently, the great progress of isolation and in vitro culture of primary intramuscular preadipocytes in livestock attracts much attention to increase its content from aspects of development and genetics [4]. Thus, the in vitro intramuscular adipocyte model makes it possible to explore the underlying mechanic regulatory network for IMF deposition.

The molecular regulators involved in process of preadipocyte adipogenesis from subcutaneous or visceral white adipose tissues (SWAT or VWAT) have been well established in recent decades, including key transcriptional factors (CCAAT enhancer binding protein, Cebpβ; Cebpα; Peroxisome proliferator activated receptor gamma, Pparg; sterol regulatory element binding protein isoform 1c, Srebp1c; and Kruppel like factor, Klf8) and triglycerides synthesis genes (Fatty acids binding protein, Fabp4 or aP2) [5]. As the specific anatomic properties of intramuscular adipocytes, dispersed inside muscle tissues and influenced by muscle growth rate and its metabolic activity, intramuscular preadipocyte displays distinctive proliferation or differentiation pattern compared to classical adipocytes [4], which parallels several lines of demotic animals. It was reported that subcutaneous preadipocytes had higher potential for proliferation and adipogenesis than those of intramuscular preadipocytes in both Bamei and Landrace pigs [6]. In addition, although RNA-seq or microarray assay explores the global comparison gene expression and miRNAome between subcutaneous and intramuscular adipocytes in pig, bovine or goat [7,8,9,10], the exact function and regulation mechanism of these differential regulators in intramuscular adipogenesis remains largely unknown.

Liver X receptor (LXR) are crucial nuclear hormone receptors, including LXRα and LXRβ, and play a vital role in cholesterol and lipid homeostasis [11,12,13]. LXRβ is expressed ubiquitously, whereas LXRα is restricted to tissues known to play important roles in lipid metabolism, such as the liver, adipose tissue, skeletal muscle, and adrenal gland. Recently, although increasing evidence points LXRα affecting animal adipose deposition and adipocyte lipid metabolism, discrepancies have been observed in different animal species, animal model statuses and distinguished functional fat depots [14,15,16,17]. For example, LXRα knockout (KO) blunted mice adipogenesis, demonstrating the positive role of LXRα in the regulation of lipid homeostasis in murine white adipocytes [14]. In contrast, another study found no differences in any of the selected markers of lipogenesis in adipose tissue specific LXRα KO compared to wild type (WT)mice on high fat diet [15]. Thus, the function of LXRα involved in goat intramuscular adipocyte adipogenesis needs to be further defined in view of the species difference and anatomic specifics.

To explore LXRα regulating adipogenic differentiation of intramuscular preadipocyte, the LXRα expression patterns were firstly detected in various tissues and during intramuscular adipogenesis. Then, the loss and gain of function for LXRα mediated by siRNAs and adenovirus in primary intramuscular preadipocyte model, respectively, were performed to reveal the role for intramuscular preadipocyte differentiation. Mechanically, we predicted the putative targets of LXRα, which was manifested by expression pattern in the process of intramuscular adipocyte differentiation and expression changes in loss function of LXRα intramuscular mature adipocytes. Taken together, our study suggests that LXRα is a positive regulator for goat intramuscular adipogenesis and provides new insights in goat quality improvement.

## 2. Results

### 2.1. The Expression Pattern of LXRα in Goat Various Tissues and During Intramuscular Adipocytes Differentiation

**Figure 1:**
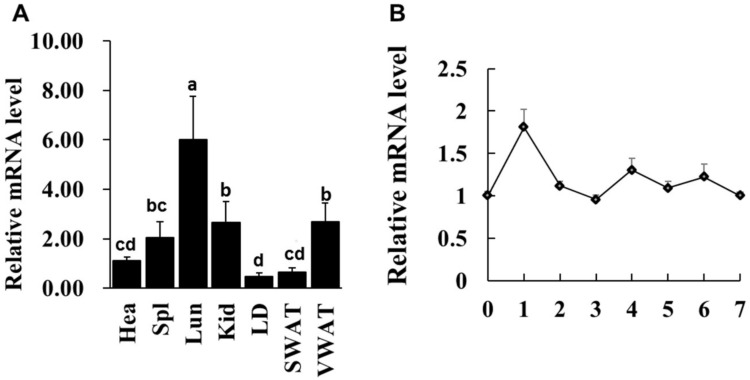
The *LXRα* gene expression pattern in various tissues and during intramuscular preadipocyte differentiation. (**A**) The *LXRα* mRNA level in heart (Hea), spleen (Spl), lung (Lun), longissimus dorsi (LD) muscle, subcutaneous white adipose tissue (SWAT) and visceral WAT (VWAT), *n* = 6. (**B**) The *LXRα* mRNA level on Days 0–7 in induced differentiation intramuscular adipocyte (*n* = 6). Data are shown as the means ± standard error of the mean (SEM). Different lowercase represents significant difference (*p* < 0.05).

Previous study reported that *LXRα* mRNA abundance is enriched in mouse lipid metabolic related tissues [11], whether this expressional characteristic in goat is consistent with that of mouse. Various tissues from one-year-old goat were collected and qPCR analysis was performed. The data showed that *LXRα* had highest mRNA level in lung (Lun), six-fold higher than heart (Hea), whereas its mRNA level was lowest in longissimus dorsi (LD) muscle and middle expression level in kidney (Kid), visceral white adipose tissue (VWAT) and spleen (Spl), three-, three- and two-fold higher than heart, respectively (Figure 1A). Given that the *LXRα* mRNA level in muscle does not completely represent its expression in IMF because of extremely small population of intramuscular adipocytes in skeletal muscle tissue, intramuscular preadipocytes were isolated from longissimus dorsi (LD) muscle and *LXRα* mRNA level was detected during intramuscular adipogenic differentiation. As shown in Figure 1B, the mRNA expression of *LXRα* reached a peak at Day 1 after differentiation and then exhibited a decrease trend from Day 2 to Day 7. These data suggest that LXR*α* might regulate intramuscular adipogenesis at early stage of differentiation.

### 2.2. Loss Function of LXRα Suppresses Intramuscular Adipocytes Differentiation

**Figure 2:**
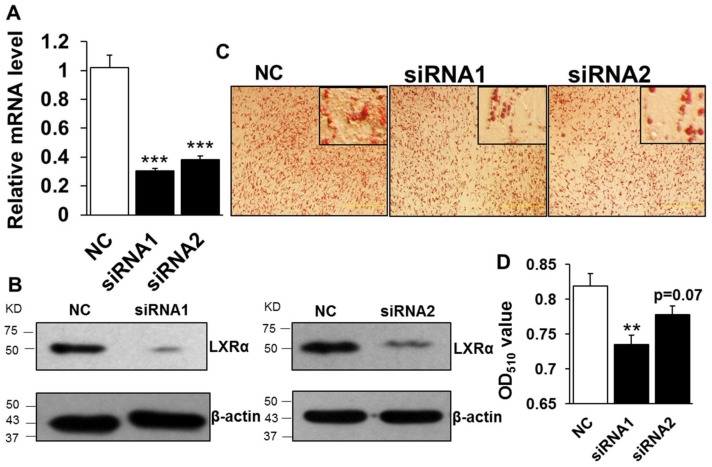
Knockdown of LXRα inhibits goat intramuscular adipocyte lipid accumulation. (**A**,**B**) The knockdown efficiency of *LXRα* at mRNA (**A**) and protein (**B**) level (*n* = 6). (**C**,**D**) The Oil Red O staining (×100) and lipid accumulation between control and siRNAs treatment intramuscular adipocyte cells (*n* = 6). ** *p* < 0.01, *** *p* < 0.001, compared to that of negative control (NC). Data are shown as the means ± SEM.

To elucidate the function of LXR*α* regulation on intramuscular lipid accumulation, we performed knockdown of LXR*α* mediated by transfecting two-independent siRNAs into preadipocytes. The knockdown efficiency assay showed that designed siRNAs decreased the mRNA level of *LXRα* significantly, by ~70% and ~60% compared to that of negative control (NC) in siRNA1 and siRNA2 treatment, respectively (Figure 2A). Consistently, the protein level of LXRα was dramatically inhibited in both siRNA1 and siRNA2 treated groups, compared to that of NC (Figure 2B). Further, the lipid accumulation in intramuscular adipocytes, caused by loss function of LXR*α*, was determined by Oil Red O staining and its extraction measurement. As shown in Figure 2C, both siRNA1 and siRNA2 treatments reduced adipocytes lipid accumulation, with fewer lipid droplets than those of NC group. Statistically, the Oil Red O signal was obviously decreased in knockdown cells mediated by siRNA1 (Figure 2D). Although the difference of Oil Red O signal between NC and siRNA2 is not significant, it still exhibited a decrease trend (Figure 2D, *p* = 0.07). These data indicated that loss function of LXR*α* inhibits intramuscular adipocytes lipid accumulation.

**Figure 3:**
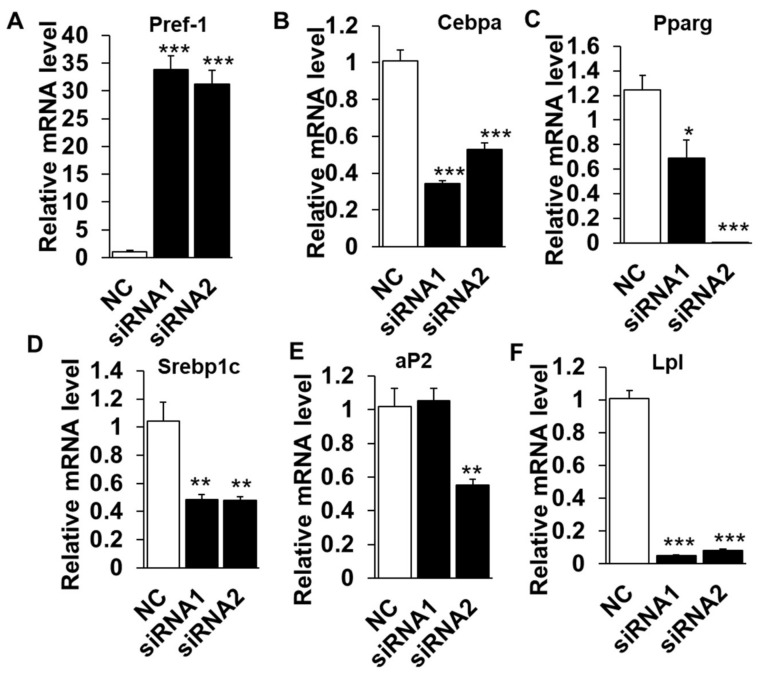
Knockdown of LXRα upregulates negative- and downregulates positive-intramuscular adipogenic genes. The mRNA levels between control and siRNAs treatment intramuscular adipocyte cells (*n* = 6) of: *Pref-1* (**A**); *Cebpα* (**B**); *Pparg* (**C**); *Srebp1c* (**D**); *aP2* (**E**); and *Lpl* (**F**). * *p* < 0.05, ** *p* < 0.01, *** *p* < 0.001, compared to that of negative control (NC). Data are shown as the means ± SEM.

The lipid deposition is a well-orchestrated multistep process that requires the sequential activation or suppression of numerous positive or negative regulators [18,19]. To determine if the reduced lipid content in LXR*α* knockdown cells is due to higher lipolysis activity or a failure to differentiate into lipid accumulating adipocytes, we examined the mRNA levels of various adipogenic, lipogenic and lipolysis genes. The mRNA level of *pref-1*(also called *Dlk1*), as a maker of preadipocytes and a negative regulator for adipogeneses, robustly increased by ~35 folds in knockdown groups, compared to that of NC (Figure 3A) and suggested that LXR*α* might affect the adipogenic differentiation initiation. Moreover, inhibition of LXR*α* down-regulated mRNA level of adipogenic related transcriptional factors, including *Cebpα* and *Pparg* (Figure 3B,C). In addition, *Srebp1c* and *aP2*, the key genes of lipogenesis and triglycerides synthesis processes, were also inhibited in siRNAs treated cells (Figure 3D,E). Interestingly, *Lpl*, a lipolysis-related gene, was with a dramatically lower mRNA level in knockdown adipocytes than that of control (Figure 3F). Collectively, these data demonstrated that knocking down of LXR*α* suppresses intramuscular adipocytes differentiation and expression of adipogenic related genes.

### 2.3. Overexpression of LXRα Does Not Affect Intramuscular Adipocytes Differentiation 

**Figure 4:**
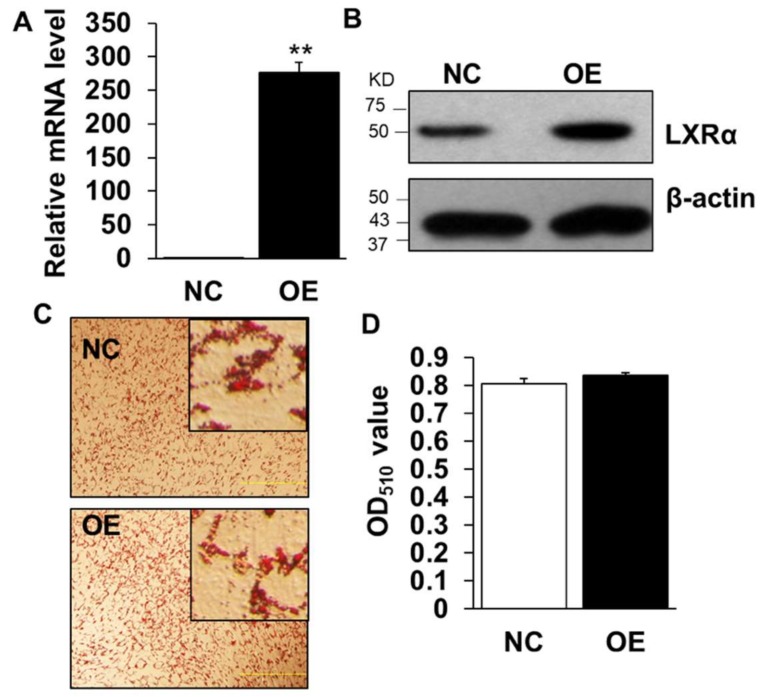
Overexpression of LXRα does not impact on goat intramuscular adipocyte differentiation. (**A**,**B**) The overexpression efficiency of *LXRα* at mRNA and protein level (*n* = 6). (**C**,**D**) The Oil Red O staining (×100) and lipid accumulation between control and overexpression treatment intramuscular adipocyte cells (*n* = 4). Data are shown as the means ± SEM.

**Figure 5:**
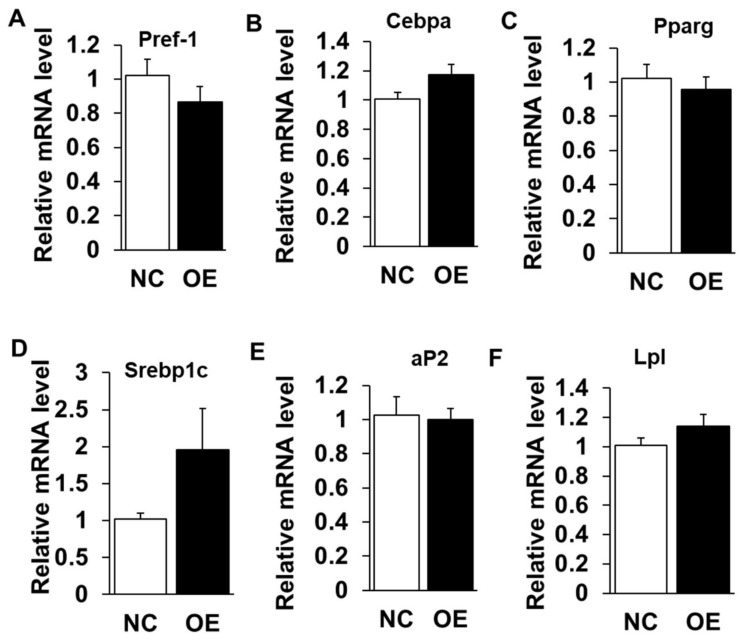
Overexpression of LXRα does not influence intramuscular adipogenic genes. (**A**–**F**) The mRNA levels between control and siRNAs treatment intramuscular adipocyte cells (*n* = 6) of: *Pref-1* (**A**) *Cebpα* (**B**); *Pparg* (**C**); *Srebp1c* (**D**); *aP2* (**E**); and *Lpl* (**F**). Data are shown as the means ± SEM.

We next performed gain-of-function analysis using adenovirus-mediated overexpression of LXR*α* in cultured intramuscular preadipocytes isolated from LD muscle. Overexpression (OE) led to ~300 times increase to endogenous *LXRα* level at Day 3 after adipogenic induction (Figure 4A), which was further confirmed by Western blot analysis (Figure 4B), OE with robustly higher LXR*α* protein level than that of control treated (Figure 4B). However, OE of LXR*α* did not affect lipid contents in intramuscular adipocytes differentiated from primary intramuscular preadipocytes, assessed by Oil Red O staining and its signal measurement (Figure 4C,D). Consistently, the mRNA level of adipogenic-related genes, including *Pref-1*, *Cebpα*, *Pparg*, *Srebp1c*, *aP2* and *Lpl*, were comparable to those of NC (Figure 5A–F). Altogether, these data demonstrated that OE of LXR*α* does not affect intramuscular adipocytes lipid content and expression of adipogenic-related genes.

### 2.4. LXRα Effects on Intramuscular Adipocytes Differentiation through Upregulation of Cebpβ and Klf8

**Figure 6:**
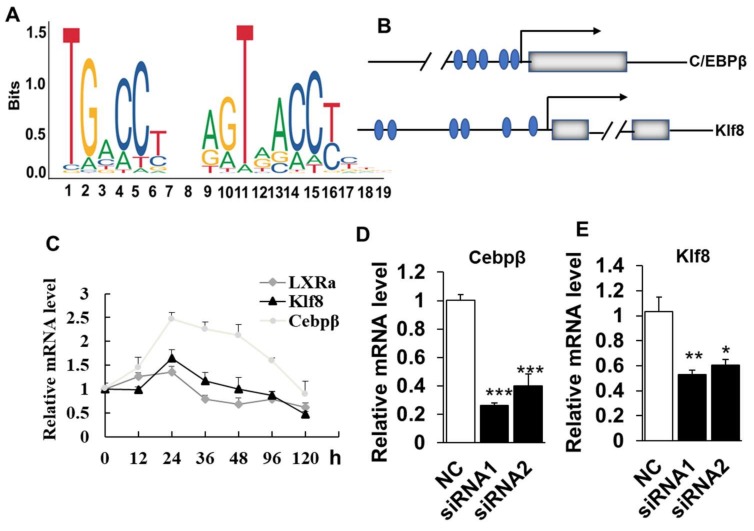
Knockdown of LXRα inhibits intramuscular adipogenesis trough downregulation of Cebpβ and Klf8: (**A**) the LXRα binding DNA motif; (**B**) the LXRα binding sites prediction at the promoters of Cebpβ and Klf8, blue circles represent the LXRα binding sites, gray boxes represent exons; (**C**) the *Cebpβ* and *Klf8* expression trend during intramuscular adipogenesis (*n* = 4); and (**D**,**E**) the mRNA levels of *Cebpβ* and *Klf8* in LXRα-siRNA treated cells (*n* = 6). Data are shown as the means ± SEM. * *p* < 0.05, ** *p* < 0.01, *** *p* < 0.001, compared to that of negative control (NC).

As LXR*α* was required for the differentiation and adipogenic-related genes expression of intramuscular adipocytes, we next sought to identify the potential downstream targets of LXR*α*. To achieve this, we first analyzed the transcriptional binding DNA motif of LXR*α* from JASPAR software (http://jaspar.genereg.net/). As shown in Figure 6A, LXR*α* binding to DNA as a heterodimer with nuclear receptor retinoid X receptor (RXR) on direct repeats (DRs) of TGACCT spaced by 4 nucleotides (DR4) [20]. We further analyzed the putative binding sites of LXRα in the promoter of adipogenic related genes, including *Cebpβ, Cebpα, Pparg, Lpl* and *Klf8*. The data showed that *Cebpβ* and *Klf8* were predicted as the potential targets of LXRα, with five and six potential binding sites in the promoter region, respectively (Figure 6B). Consistently, the mRNA expressional patterns of *Cebpβ* and *Klf8* were in line with that of LXRα during intramuscular adipogenesis (Figure 6C). As our speculation, loss function of LXRα also downregulated mRNA levels of *Cebpβ* and *Klf8* significantly in goat intramuscular adipocyte (Figure 6D,E). Altogether, these data suggest that LXRα affects intramuscular adiposity might through regulation of both Cebpβ and Klf8. 

## 3. Discussion

Understanding regulators that affect intramuscular adipogenesis may lead to identification of candidates to appropriately increase IMF content to improve the goat meat. Here, we report that LXRα is a positive regulator for intramuscular adipocyte differentiation, which is proven by knockdown of LXRα decreasing adipocyte lipid accumulation and adipogenic related genes expression. Mechanically, LXRα might regulate Cebpβ and Klf8 to be involved in intramuscular adiposity. Our research expands the growing knowledge of regulation network of goat intramuscular fat deposition.

Previous research reported that LXRα is mainly expressed in organs involved in lipid metabolism such as intestine, adipose tissue, and macrophages [12]. In this study, we found highest level of *LXRα* in lung tissue, which suggests LXRα may be required for maintaining lung’s function. This is supported by ablation of Liver X receptors α and β leading to spontaneous peripheral squamous cell lung cancer in mice [21]. In adipose tissue, the *LXRα* expression in VWAT is higher than that of in SWAT and this expression pattern could be explained by visceral adipocyte with larger capacity of lipid storage than subcutaneous adipocyte [22]. Beyond our expectation, the LD (containing IMF) had the lowest mRNA level of *LXRα* among detected tissues. We speculate that this might be contaminated by a large number of myofibers and just small proportion intramuscular adipocytes in whole LD muscle [23,24,25]. Intriguingly, the LXRα expression trend during isolated intramuscular differentiation confirms that LXRα possibly affects intramuscular adipogenic process at early stage. However, the mRNA level of *LXRα* was highest at Day 12 after inducing differentiation in human SGBS preadipocytes [26]. We speculate that this might be supported by the difference the experimental cells.

Our data show that LXRα is necessary for intramuscular lipid accumulation and expression of adipogenic related genes, which is supported by several lines. Firstly, both two independent siRNAs knocking down of LXRα reduces intramuscular adipocytes lipids content and indicates this role is not caused by off target effect. Moreover, loss-function-of LXRα robustly promotes the adipogenic negative regulator expression, such as *Pref1* [27]. In addition, Pref1 is identified as a preadipocytes maker [26] and this change induced by LXRα knockdown is consistent with highest *LXRα* level at early stage during intramuscular adipogenic differentiation. Conversely, disruption of LXRα suppresses the adipogenic positive regulators, including *Cebpα*, *Pparg*, *Srebp1c* and *aP2*. Among these factors, Pparg and Cebpα have been established as essential components of transcriptional cascades that precede the formation of mature adipocytes [28,29]. Interestingly, the lipolysis gene-*Lpl* had a decrease level in knockdown cells, indicating that inhibition of LXR*α* reducing the lipids accumulation is major caused by triglycerides synthesis reduction, which is also manifested by lower mRNA level of *Srebp1c* and *aP2* in siRNA treated cells. In our study, infection of LXRα adenovirus into isolated intramuscular adipocytes results in both significant increase in mRNA and protein level of LXRα. However, this overexpression does not significantly enhance the intramuscular adipogenesis and expression of adipogenic specific genes even if some adipogenic genes have a promotion trend. It is the discrepancy that overexpression LXRα in mouse mesenchymal stem cells (MSCs) inhibits adipocyte differentiation [30] and LXRα activated by LXR agonist (T0901317) in 3T3-L1 cells [31] promotes adipogenesis process. The distinct effect of LXRα OE in intramuscular adipocyte might be a result of difference of animal species and cell types. Moreover, this relatively small response of intramuscular adipocytes to LXRα overexpression can be explained by the high endogenous levels of LXRα in intramuscular adipocytes, whose Ct value is nearly comparable to internal control-*Ppia*. In addition, it is reported that LXRα functions by forming obligate heterodimers with the retinoid X receptor α (RXRα), and subsequently binds to specific DNA response elements within the regulatory regions of their target genes [32,33]. Alternatively, the small responses in overexpression cells might be explained by absence of enough RXRα to form heterodimers to bind targets.

We identified two potential target genes of LXRα in intramuscular adipogenic differentiation by bioinformatic analysis, including Cebpβ and Klf8, which is also confirmed by similar expression pattern among them during IMF adipogenesis and dramatic decrease of both *Cebpβ* and *Klf8* mRNA level in loss-function of intramuscular fat cell. In classical adipose tissue, such as subcutaneous WAT, Apolipoprotein E (ApoE) and Srebp1c are reported to be targets of LXRα in reverse cholesterol transport [34,35]. Interestingly, previous research reports that *Lpl* gene is a direct target of LXRα in the liver and macrophages, but not in adipose tissue and muscles. Consistently, we did not predict the potential binding sites of LXRα in the promoter of goat *Lpl* gene. These suggest that function and mechanism of LXRα emerge distinctively in different tissues and species. Although the *Lpl* expression is also inhibited significantly in knockdown cells, this might be a result of secondary effect from LXRα disruption cells. In other research, activated LXR induces transcriptional expression of *Pparg* to stimulate adipocyte differentiation [31]. However, promoter of goat *Pparg* did not contain predicted the binding sites of LXRα and decreased mRNA level of *Pparg* may be secondary effects caused by Cebpβ and Klf8. Thus, we speculate that knockdown of LXRα might through downregulating the transcriptional expression of *Cebpβ* and *Klf8* and subsequently results in reduction of middle-terminal adipogenic genes level and lipid accumulation in intramuscular adipocyte.

In conclusion, we report that LXRα is required for goat intramuscular adipocyte lipid deposition and adipogenic genes expression. Mechanically, *Cebpβ* and *Klf8* are identified as potential target genes of LXRα during this process. These results not only expand the understanding of regulation network of IMF deposition, but also suggest that LXRα may represent a new target in improvement of goat meat quality.

## 4. Materials and Methods 

### 4.1. Animal and Cell Culture

Animal studies were approved by the Animal Care and Use Committee of Southwest Minzu University and the Animal Disease Control Center of Sichuan province, China. The experimental animal certification number was SYXK2011-043 [36]. The seven-day-old Jianzhou Daer goat (Capra hircus) was purchased from Sichuan Jianyang Dageda Aminal Husbandry Co., Ltd. (Sichuan, China). The goat intramuscular preadipocytes isolation was performed as previous described [37]. In brief, longissimus dorsi was isolated from slaughtered seven-day-old Jianzhou Daer goat under sterile conditions, washed twice in phosphate buffered solution (PBS) supplemented with 1% penicillin/streptomycin and then minced into 1 mm^3^ pellets. Enzymatic digestion was performed with 0.2% collagenase type II (Sigma, St. Louis, Missouri, USA) at 37 °C in the water bath for 1 h with gentle agitation and terminated by the same volume of DMEM/F12 (Hyclone, Logan, Utah, USA) supplemented with 10% FBS (fetal bovine serum). The suspension was filtered on a 75 μm nylon cell strainer and centrifuged at 2000 r/min for 5 min. After disposing of the red blood cell lysed solution, the suspension was centrifuged at 2000 r/min for 5 min again and the pre-adipocytes were re-suspended in DMEM/F12 supplemented with 10% FBS and diluted to a final concentration of 10^6^ cells/mL. These cells were cultured at 37 °C in a humidified atmosphere containing 5% CO_2_.

### 4.2. Adenovirus Generation

The adenovirus with LXRα insertion was generated using the AdEasy system as described [38]. The CDS sequence of goat LXRα was cloned to pAdTrack-CMV, named pAdTrack-CMV-LXRα. The linearized pAdTrack-CMV-LXRα by restriction enzyme Pme Ι and plasmid BJ5183 were co-transformed into DH5α (Tiangen, China) for homologous recombination. Recombinant adenovirus plasmid was screened by restriction enzyme Pac I digest and then packaged adenovirus in HEK293A cells by Lipofectamine TM 3000. After 2 weeks, the recombinant adenovirus was collected by three freezes–thaw–vortex cycles. Two more round infected HEK293A cells were adapted to amplify the recombinant virus and the titers were determined by the expression of GFP. Ad-GFP, as the control, is stored in our laboratory. 

### 4.3. Chemical Synthesis of siRNA

Two gene specific siRNA for LXRα were designed online (https: //rnaidesigner. invitrogen.com/rnaiexpress/) and synthesized according to the sequence of goat LXRα (NM_001285751.1), named LXRα siRNA-1 (5’-CAUGCGGGAGGAGUGUGUCUUAUCA-3’) and LXRα siRNA-2 (5’-AUAACUGAAAUCCUUGAGGAAGGUG-3’). Negative control was provided by Invitrogen (5′-UUCUCCGAACGUGUCACGUTT-3′). 

### 4.4. Cell Induction, Transfection and Infection

The goat intramuscular preadipocytes reached 80% confluence and were adipogenic induced by DMEM/F12 containing 10% FBS and 100 μM oleic acid (Sigma, St. Louis, Missouri, USA) as described [39]. siRNA transfection was performed by Lipofectamine^®^ RNAIMAX Reagent (Invitrogen, Karlsruhe, Germany) at 70–80% preadipocytes confluence. Then, cells were analyzed by qPCR and Oil Red O staining at Day 4 after adipogenic induction. For overexpression, Ad-GFP (negative control, NC) or Ad-LXRα was used to infect cells, which were collected and monitored at day 4 after adipogenic differentiation.

### 4.5. Oil Red O Staining

Cultured cells were washed with PBS and fixed with 4% formaldehyde for 15 min at room temperature. Then the cells were stained using the Oil Red O working solutions containing 6 mL Oil Red O stock solution (5 g/L in isopropanol) and 4 mL ddH_2_O for 20 min. After staining, the cells were washed with 60% isopropanol in PBS and pictured using an Olympus TH4-200 microscope (Tokyo, Japan) with the 10X objective (NA 0.70) for higher magnification views. Oil Red O dye was extracted from stained adipocytes with 100% isopropanol, and the Oil Red signals were quantified by measuring the optical density at 490 nm (OD 490).

### 4.6. Total RNA Extraction and Quantitative Real-Time PCR (qPCR)

Total RNA was extracted from cultured cell samples or tissues by using Trizol reagent (Takara, Dalian, China) and treated with RNase-free DNase (Tiangen, China) at 42 °C for 3 min to remove genomic DNA contamination. The integrity of the total RNA was detected by 2% agarose gel electrophoresis and the concentration was determined by using ultraviolet spectrophotometer (BioSpec-nano, Shimadzu, Kyoto, Japan). For each of cell sample, 1 μg of total RNA was reverse transcribed by RevertAid First Strand cDNA Synthesis Kit (Thermo, Waltham, MA, USA) according to the manufacturer. Peptidylproyl isomerase A (*Ppia*) was selected to normalize the expression levels. The primer information for qPCR is listed in Table 1. SYBR^®^ Premix Ex Taq TM (2×) (Takara, Dalian, China) and CFX96 (Bio-Rad, Hercules, CA, USA) were used to perform qPCR. The 2^−ΔΔ*C*t^ method was used to analyze the relative mRNA level of each of genes.

### 4.7. Protein Extraction and Western Blot Analysis

Total protein was isolated from tissues using RIPA buffer contains 50 mM Tris-HCl (pH 8.0), 150 mM NaCl, 1% NP-40, 0.5% sodium deoxycholate and 0.1% SDS. Protein concentrations were measured using BCA Protein Assay Reagent (Thermo scientific, Waltham, MA, USA). Proteins were separated by sodium dodecyl sulfate polyacrylamide gel electrophoresis (SDS-PAGE), transferred to a polyvinylidene fluoride (PVDF) membrane (Millipore Corporation, Bedford, MA, USA), blocked in 5% fat-free milk for 45 min at room temperature, and then incubated with first antibodies (diluted in 5% milk) overnight at 4 °C. LXRα and β-actin antibodies are from Abcam (Cambridge, MA, USA) (ab176323, 1:2000) and Bioss (Beijing, China) (bs-10966R, 1:2000) companies, respectively. The horseradish peroxidase (HRP)-conjugated secondary antibody (anti-rabbit IgG, 111-035-003 or anti-mouse IgG, 115-035-003, Jackson ImmunoResearch, Grove, PA, USA) was diluted 1:10,000. Immunodetection was performed using enhanced chemiluminescence Western blotting substrate (Santa Cruz biotechnology, CA, USA) and detected by ChemiDoc XRS system (Bio-Rad, Hercules, CA, USA). 

### 4.8. Statistical Analysis

All the data are given as “means ± SEM”. Analysis of variance in SPSS was used to compare significance, followed by unpaired two-tailed Student’s *t*-tests. *p* < 0.05 was considered significant difference. 

## Figures and Tables

**Table 1:** Primers for quantitative real-time PCR (qPCR).

Gene	Sequence (5’-3’)	Sequence (5’-3’)	GenBank
*Cebpα*	CCGTGGACAAGAACAGCAAC	AGGCGGTCATTGTCACTGGT	XM_018062278.1
*LXRα*	TCGGAGGTACAACCCTGGAA	ATGGCAATGAGCAAGACAAACT	NM_001285751.1
*Cebpβ*	CAAGAAGACGGTGGACAAGC	AACAAGTTCCGCAGGGTG	XM_018058020.1
*Srebp1c*	AAGTGGTGGGCCTCTCTGA	GCAGGGGTTTCTCGGACT	NM_001285755.1
*Klf8*	GACTACAGCAAGAACCAGCAGC	CTCCTGTATGGATTCTGCGGT	KX247671
*aP2*	TGAAGTCACTCCAGATGACAGG	TGACACATTCCAGCACCAGC	NM_001285623.1
*Lpl*	TCCTGGAGTGACGGAATCTGT	GACAGCCAGTCCACCACGAT	NM_001285607.1
*Pparg*	AAGCGTCAGGGTTCCACTATG	GAACCTGATGGCGTTATGAGAC	NM_001285658.1
*Pref1*	CCGGCTTCATGGATAAGACCT	GCCTCGCACTTGTTGAGGAA	KP686197.1
*Ppia*	ACAAAGTCCCGAAGACAGCAG	AAGTCACCACCCTGGCACAT	XM_005679322.2

## Abbreviations

IMF	Intramuscular fat
LXRα	Liver X receptor α
Cebpα	CCAAT enhancer binding protein α
Cebpβ	CCAAT enhancer binding protein β
Pparg	Peroxisome proliferator activated receptor gamma
Srebp1c	Sterol regulatory element binding protein isoform 1c
Klf8	Kruppel like factor 8
aP2	Fatty acids binding protein 4
Lpl	Lipoprotein lipase
SWAT	Subcutaneous white adipose tissue
VWAT	Visceral white adipose tissues
KO	Knockout
WT	Wild type
LD	Longissimus dorsi
NC	Negative control
RXRα	Retinoid X receptor α
ApoE	Apolipoprotein E

## References

[1] Fernandez X., Monin G., Talmant A., Mourot J., Lebret B. (1999). Influence of intramuscular fat content on the quality of pig meat-2. Consumer acceptability of m. longissimus lumborum. Meat Sci..

[2] Cheng W.W., Cheng J.H., Sun D.W., Pu H.B. (2015). Marbling Analysis for Evaluating Meat Quality: Methods and Techniques. Compr. Rev. Food Sci. Food Saf..

[3] Addison O., Marcus R.L., LaStayo P.C., Ryan A.S. (2014). Intermuscular Fat: A Review of the Consequences and Causes. Int. J. Endocrinol..

[4] Hocquette J.F., Gondret F., Baeza E., Medale F., Jurie C., Pethick D.W. (2010). Intramuscular fat content in meat-producing animals: Development, genetic and nutritional control, and identification of putative markers. Animal.

[5] Lowe C.E., O’Rahilly S., Rochford J.J. (2011). Adipogenesis at a glance. J. Cell Sci..

[6] Zhang G.H., Lu J.X., Chen Y., Zhao Y.Q., Guo P.H., Yang J.T., Zang R.X. (2014). Comparison of the adipogenesis in intramuscular and subcutaneous adipocytes from Bamei and Landrace pigs. Biochem. Cell Biol..

[7] Sun W.X., Wang H.H., Jiang B.C., Zhao Y.Y., Xie Z.R., Xiong K., Chen J. (2013). Global comparison of gene expression between subcutaneous and intramuscular adipose tissue of mature Erhualian pig. Genet. Mol. Res..

[8] Du C., Fu S.Y., Gao H.Y., Zheng Z.Q., Meng X.R., A N., Sa R., Zhang W.G., Li J.Q. (2014). Transcriptome Analysis of Intramuscular Preadipocytes and Matureadipocyte in Cashmere Goats. Acta Veterinaria et Zootechnica Sinica.

[9] Sheng X.H., Ni H.M., Liu Y.H., Li J.Y., Zhang L.P., Guo Y. (2014). RNA-seq analysis of bovine intramuscular, subcutaneous and perirenal adipose tissues. Mol. Biol. Rep..

[10] Guo Y.X., Mo D.L., Zhang Y., Zhang Y., Cong P.Q., Xiao S.Q., He Z.Y., Liu X.H., Chen Y.S. (2012). MicroRNAome Comparison between Intramuscular and Subcutaneous Vascular Stem Cell Adipogenesis. PLOS ONE.

[11] Schulman I.G. (2017). Liver X receptors link lipid metabolism and inflammation. FEBS Lett..

[12] Korach-Andre M., Gustafsson J.A. (2015). Liver X receptors as regulators of metabolism. Biomol. Concepts.

[13] Hong C., Tontonoz P. (2014). Liver X receptors in lipid metabolism: opportunities for drug discovery. Nat. Rev. Drug Discov..

[14] Archer A., Laurencikiene J., Ahmed O., Steffensen K.R., Parini P., Gustafsson J.A., Korach-Andre M. (2014). Skeletal muscle as a target of LXR agonist after long-term treatment: focus on lipid homeostasis. Am. J. Physiol.-Endoc. Metab..

[15] Dib L., Bugge A., Collins S. (2014). LXRα fuels fatty acid-stimulated oxygen consumption in white adipocytes. J. Lipid Res..

[16] Ulven S.M., Dalen K.T., Gustafsson J.A., Nebb H.I. (2005). LXR is crucial in lipid metabolism. Prostag. Leukot. Ess..

[17] Stenson B.M., Ryden M., Venteclef N., Dahlman I., Pettersson A.M., Mairal A., Astrom G., Blomqvist L., Wang V., Jocken J.W. (2011). Liver X receptor (LXR) regulates human adipocyte lipolysis. J. Biol. Chem..

[18] Cohen P., Spiegelman B.M. (2016). Cell biology of fat storage. Mol. Biol. Cell.

[19] Ali A.T., Hochfeld W.E., Myburgh R., Pepper M.S. (2013). Adipocyte and adipogenesis. Eur. J. Cell Biol..

[20] Khan A., Fornes O., Stigliani A., Gheorghe M., Castro-Mondragon J.A., van der Lee R., Bessy A., Cheneby J., Kulkarni S.R., Tan G. (2018). JASPAR 2018: update of the open-access database of transcription factor binding profiles and its web framework. Nucleic Acids Res.

[21] Dai Y.B., Miao Y.F., Wu W.F., Li Y., D’Errico F., Su W., Burns A.R., Huang B., Maneix L., Warner M. (2016). Ablation of Liver X receptors alpha and beta leads to spontaneous peripheral squamous cell lung cancer in mice. Proc. Natl. Acad. Sci. USA.

[22] Ibrahim M.M. (2010). Subcutaneous and visceral adipose tissue: structural and functional differences. Obes. Rev..

[23] Yang X.J., Albrecht E., Ender K., Zhao R.Q., Wegner J. (2006). Computer image analysis of intramuscular adipocytes and marbling in the longissimus muscle of cattle. J. Anim. Sci..

[24] Hausman G.J., Basu U., Du M., Fernyhough-Culver M., Dodson M.V. (2014). Intermuscular and intramuscular adipose tissues: Bad vs. good adipose tissues. Adipocyte.

[25] Aldai N., Najera A.I., Dugan M.E., Celaya R., Osoro K. (2007). Characterisation of intramuscular, intermuscular and subcutaneous adipose tissues in yearling bulls of different genetic groups. Meat. Sci..

[26] Lahnalampi M., Heinäniemi M., Sinkkonen L., Wabitsch M., Carlberg C. (2010). Time-resolved expression profiling of the nuclear receptor superfamily in human adipogenesis. PLoS ONE.

[27] Wang Y.H., Kim K.A., Kim J.H., Sul H.S. (2006). Pref-1, a preadipocyte secreted factor that inhibits adipogenesis. J. Nutr..

[28] Kim S.W., Muise A.M., Lyons P.J., Ro H.S. (2001). Regulation of adipogenesis by a transcriptional repressor that modulates MAPK activation. J. Biol. Chem..

[29] Wu Z., Puigserver P., Spiegelman B.M. (1999). Transcriptional activation of adipogenesis. Curr. Opin. Cell Biol..

[30] Matsushita K., Morello F., Zhang Z., Masuda T., Iwanaga S., Steffensen K.R., Gustafsson J.Å., Pratt R.E., Dzau V.J. (2016). Nuclear hormone receptor LXRα inhibits adipocyte differentiation of mesenchymal stem cells with Wnt/beta-catenin signaling. Lab. Invest..

[31] Seo J.B., Moon H.M., Kim W.S., Lee Y.S., Jeong H.W., Yoo E.J., Ham J., Kang H., Park M.G., Steffensen K.R. (2004). Activated liver X receptors stimulate adipocyte differentiation through induction of peroxisome proliferator-activated receptor gamma expression. Mol. Cell Biol..

[32] Yue L., Ye F., Gui C., Luo H., Cai J., Shen J., Chen K., Shen X., Jiang H. (2005). Ligand-binding regulation of LXR/RXR and LXR/PPAR heterodimerizations: SPR technology-based kinetic analysis correlated with molecular dynamics simulation. Protein Sci..

[33] Willy P.J., Mangelsdorf D.J. (1997). Unique requirements for retinoid-dependent transcriptional activation by the orphan receptor LXR. Genes Dev..

[34] Laffitte B.A., Repa J.J., Joseph S.B., Wilpitz D.C., Kast H.R., Mangelsdorf D.J., Tontonoz P. (2001). LXRs control lipid-inducible expression of the apolipoprotein E gene in macrophages and adipocytes. Proc. Natl. Acad. Sci. USA.

[35] Repa J.J., Liang G., Ou J., Bashmakov Y., Lobaccaro J.M., Shimomura I., Shan B., Brown M.S., Goldstein J.L., Mangelsdorf D.J. (2000). Regulation of mouse sterol regulatory element-binding protein-1c gene (SREBP-1c) by oxysterol receptors, LXRα and LXRβ. Genes Dev..

[36] Zhao Y., Li R., Lin Y. (2015). Allograft inflammatory factor-1 in grass carp (Ctenopharynogodon idella): Expression and response to cadmium exposure. Fish Shellfish Immunol..

[37] Chen F.F., Xiong Y., Peng Y., Gao Y., Qin J., Chu G.Y., Pang W.J., Yang G.S. (2017). miR-425-5p Inhibits Differentiation and Proliferation in Porcine Intramuscular Preadipocytes. Int. J. Mol. Sci..

[38] Xiong Y., Yue F., Jia Z., Gao Y., Jin W., Hu K., Zhang Y., Zhu D., Yang G., Kuang S. (2018). A novel brown adipocyte-enriched long non-coding RNA that is required for brown adipocyte differentiation and sufficient to drive thermogenic gene program in white adipocytes. Biochim. Biophys. Acta.

[39] Shang Z., Guo L., Wang N., Shi H., Wang Y., Li H. (2014). Oleate promotes differentiation of chicken primary preadipocytes in vitro. Bioscience rep..

